# Depression From a Precision Mental Health Perspective: Utilizing Personalized Conceptualizations to Guide Personalized Treatments

**DOI:** 10.3389/fpsyt.2021.650318

**Published:** 2021-05-11

**Authors:** Reem Deif, Mohamed Salama

**Affiliations:** ^1^Institute of Global Health and Human Ecology, School of Sciences and Engineering, The American University in Cairo, Cairo, Egypt; ^2^Faculty of Medicine, Mansoura University, Mansoura, Egypt; ^3^Global Brain Health Institute, Trinity College Dublin, Dublin, Ireland

**Keywords:** depression, neural circuitry, psychosocial markers, biomarkers, precision mental health

## Abstract

Modern research has proven that the “typical patient” requiring standardized treatments does not exist, reflecting the need for more personalized approaches for managing individual clinical profiles rather than broad diagnoses. In this regard, precision psychiatry has emerged focusing on enhancing prevention, diagnosis, and treatment of psychiatric disorders through identifying clinical subgroups, suggesting personalized evidence-based interventions, assessing the effectiveness of different interventions, and identifying risk and protective factors for remission, relapse, and vulnerability. Literature shows that recent advances in the field of precision psychiatry are rapidly becoming more data-driven reflecting both the significance and the continuous need for translational research in mental health. Different etiologies underlying depression have been theorized and some factors have been identified including neural circuitry, biotypes, biopsychosocial markers, genetics, and metabolomics which have shown to explain individual differences in pathology and response to treatment. Although the precision approach may prove to enhance diagnosis and treatment decisions, major challenges are hindering its clinical translation. These include the clinical diversity of psychiatric disorders, the technical complexity and costs of multiomics data, and the need for specialized training in precision health for healthcare staff, besides ethical concerns such as protecting the privacy and security of patients' data and maintaining health equity. The aim of this review is to provide an overview of recent findings in the conceptualization and treatment of depression from a precision mental health perspective and to discuss potential challenges and future directions in the application of precision psychiatry for the treatment of depression.

**Graphical Abstract:**
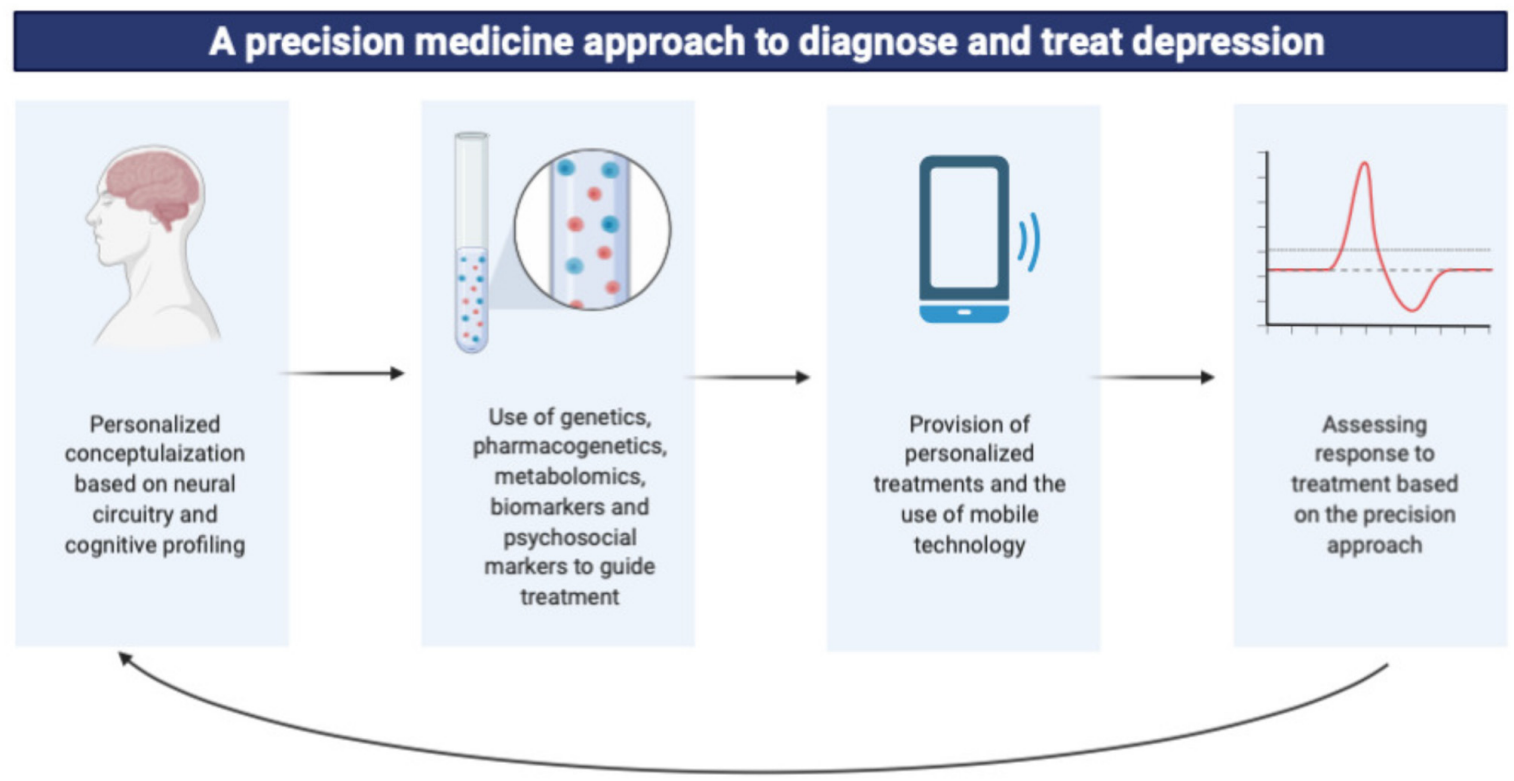
A summary of the precision medicine approach in the diagnosis and treatment of major depressive disorder using multi-omics data.

## Key Concepts

**Figure 1:**
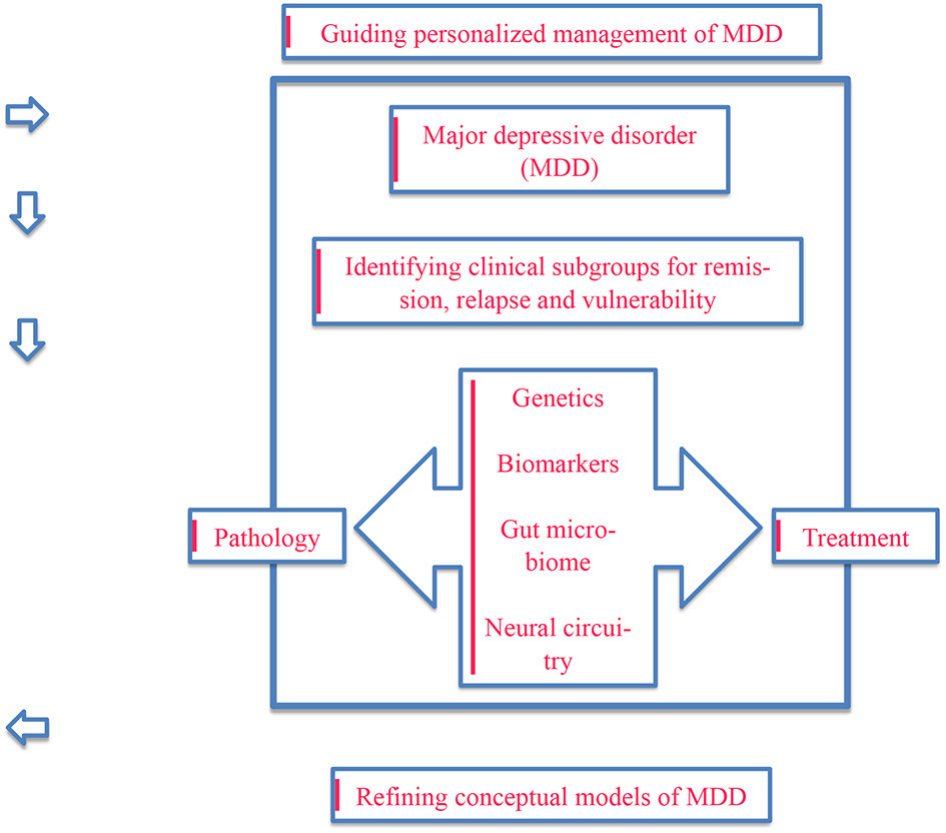
An illustration of the key concepts focusing on the need for conceptualizing and treating major depressive disorder from a precision psychiatry perspective using multiomics data.

This paper aimed to provide an overview of the implementation of precision psychiatry in the treatment of major depressive disorder using multiomics data. Key concepts are summarized in Figure 1.

## Brief Historical Perspective

Research has substantially examined various risk and protective factors in relation to psychiatric disorders besides evidence-based treatments. With more advances and insights into the pathology of different disorders, a new concept of precision medicine has emerged. According to Fernandes et al. (1), precision psychiatry is a branch of precision medicine focused on enhancing prevention, diagnosis, and treatment of psychiatric disorders through identifying clinical subgroups in relation to specific disorders, identifying subgroups that may benefit from specific disorders, assessing the effectiveness of different interventions with different subgroups, and identifying risk and protective factors for remission, relapse, and vulnerability (1).

This approach is in line with the recommendations of Insel, former director of the National Institute of Mental Health (2) who highlighted the need for precision medicine for mental disorders and, for that goal, the integration of data covering different biopsychosocial aspects when considering risk and vulnerability of an individual. However, in order to achieve the ultimate goal of personalizing mental health, there is a need for comprehensive translational research that include data from clinical trials, routine care, and cohort studies.

For example, the newly established Center for Precision Mental Health and Wellness at Stanford University aims to bridge the knowledge gap between brain sciences and mental health based in an attempt to come up with effective, evidence-based translational approaches in assessment, diagnosis, and treatment of mental disorders, more specifically depression. This is based on the examination and classification of malfunctioning brain circuitry using advanced imaging technologies. Other mental health initiatives have taken a similar path toward the enhancement of the effectiveness of mental health services through the translation of neuroscience into clinical practice, especially with increasing prevalence of mental disorders, and therefore the demands for effective services, worldwide.

Depression, diagnostically recognized as major depressive disorder, is a major health concern affecting the lives of more than 264 million people worldwide and contributing to the disability and global burden of disease. With increasing and alarming global prevalence, it has become one of the priorities covered by WHO's mental health Gap Action Programme (mhGAP) calling for “a comprehensive, coordinated response to mental disorders at the country level” as per the 2013 World Health Assembly resolution (3). The aim of this review is to provide an overview of reported findings in the understanding of the pathology and treatment of depression from a precision mental health perspective and to report on potential challenges of the implementation of precision psychiatry in the treatment of depression.

## Summary of the Established Principles

Earlier studies have attempted to show neural circuits underlying the pathogenesis of different manifestations of negative affective states in animal models (4) and have therefore inspired more recent neuroimagining research focusing on the neural circuitry of the depressed human brain. This is in line with the National Institute of Mental Health (NIMH) calling for mapping “the connectomes for mental illness” (NIMH's Strategic Plan, Objective 1, Strategy 1.3). Such endeavors have led to the conceptualization of depression as diseased brain networks rather than diseased brain regions reflecting the involvement of different neurotransmitter systems and different mechanisms in its pathology (5, 6).

Traditionally, depressive disorders are hypothesized to be directly linked with the processing of reward and punishment within different neural circuits (7). Disruptions in the topological organization of reward circuits have shown to predict severity of depressive symptoms and cognitive deficits associated with depression (8). More recently, studies have established more specific neural circuits in relation to depression, including treatment resistant depression (9).

Another ground-breaking approach toward conceptualizing depression is through identifying specific disease-related neural circuits building up on current knowledge of the large-scale circuits responsible for various cognitive and emotional processes. This is based on the assumption that functional and structural connectivity dysfunctions underlie specific “biotypes” of mental disorders. In this regard, it is argued that depression cannot be explained in terms of one pathological mechanism. For example, six neural circuit dysfunctions that have proven to be influential in both depression and anxiety have been identified in terms of their role in moderating salience, negative affect, positive affect, attention, and cognitive control in addition to the default mode (10).

Theoretical symptomatic biotypes based on six neural circuits are also proposed; these include rumination, anxious avoidance, negative bias, threat dysregulation, anhedonia, context insensitivity, inattention, and cognitive dyscontrol. In relation to precision medicine, such neural circuit biotypes can ideally guide personalized treatments for higher effectiveness. For instance, based on neural circuitry, it is suggested that patients with the negative affect biotype might mostly benefit from cognitive behavioral therapies compared to patients with the salience biotype who might mostly benefit from selective serotonin reuptake inhibitors and/or deep brain stimulation. In order to develop such an approach, use of quantitative metrics is recommended in order to categorize subgroups of patients fitting in each biotype and to identify symptoms in relation to neural circuit dysfunctions (10).

## Current State of the Art

### Genetics

Early researchers have attempted to demonstrate a link between genetics and mental disorders and in 1973, Gottesman and Shields introduced the concept of “endophenotype” in an attempt to categorize psychiatric symptoms into specific phenotypes hypothesized to be influenced by genetics. Neuropattern, a new concept used in diagnosis based on the idea of endophenotypes, was then proposed. One exploratory study showed its potential in showing a greater reduction in symptomatic severity and faster response to treatment in the Neuropattern group compared to standard care (11). Such approaches are based on the genetic variations underlying differences in brain circuitry which can explain the differences in affective, cognitive, and behavioral symptoms. With more advances in methodologies, it is estimated that genetics account for up to 42% of variations in response to antidepressants (12). Current knowledge about the role of some genes in the depression and response to treatment is growing quickly; however, most established findings are related to the pharmacokinetic genes, *CYP2D6* and *CYP2C19* (13) on which selective serotonin-reuptake inhibitors dosing guidelines are based (14). A recent review provided a summary of genetic studies examining specific single nucleotide polymorphisms (SNPs) that are relevant to response to antidepressants. These include *COMT, HTR2A, HTR1A, CNR1, SLC6A4, NPY, MAOA, IL1B, GRIK4, BDNF, GNB3, FKBP5, CYP2D6, CYP2C19*, and *ABCB1* (15). It has also been demonstrated that multiple gene modules could represent cortical dysfunction in different disorders including major depressive disorder (16). In terms of its mechanism of action, such genes are involved in the up regulation of the hypothalamic-pituitary-adrenal (HPA) axis, therefore, contributing to the development of depressive symptoms. Looking into geneX environment interactions, preliminary research also suggests the potential use of epigenetics as biomarkers for predicting response to electroconvulsive therapy (ECT) which is commonly used in treatment-resistant and severe cases of depression (17).

### Biomarkers

From an inflammatory perspective, depression can be regarded as aa pro-inflammatory condition marked by the different inflammatory biomarkers such as interleukin IL-6, TNFα, and C reactive protein (18). An alternative formulation of depression has been proposed building on the neurotrophic hypothesis which conceptualizes depression in terms of pathological alternations of growth factor proteins. One study aimed to examine the role of growth factor proteins in inpatients with treatment-resistant depression specifically (19). Blood analysis was done comparatively between patients and healthy controls to examine serum biomarkers. Findings suggest that, compared to healthy controls, patients had lower serum levels of brain-derived neurotrophic factors and vascular endothelial growth factor-C on one hand, and higher angioporetin-1 receptor biomarkers on the other hand. Additionally, and compared to patients who showed improvements in symptomatology, higher levels of vascular endothelial growth factor-D, and lower levels of vascular endothelial growth factor-C and F predicted treatment resistance in non-responders.

### Psychosocial Markers

From a cognitive perspective, research has identified specific cognitive biases involved in the processing of affective information which can be used as indicators for specific symptomatology in mood disorders, including depression. This is in reference to “hot” cognitive processes, which are emotionally-loaded, as opposed to “cold” emotion-independent cognitions. For example, patients with major depressive disorder tend to have significant cognitive biases that contribute to their symptoms in addition to slow responses, poor response to positive stimuli, heightened sensitivity to negative stimuli, and a tendency to make negative interpretations of ambiguous stimuli (20). As discussed earlier, these biases can be linked with biological models of mood disorders. Research also suggests that such hot cognitive affective abnormalities can predict response to psychopharmacological treatment and that therefore they can be used as predictors of treatment efficacy in themselves (21, 22). According to Cotter and Barnett (23), such models can be used in translational advances such as identifying, managing at-risk individuals, and guiding treatment. For example, patients who do not have such cognitive biases are less likely to benefit from cognitive behavioral therapies that aims for cognitive restructuring for the alleviation of symptoms. They may still benefit from other interventions, either psychopharmacological or psychotherapeutic, that may target the symptomatology is this specific subgroup in order to optimize treatment.

Another systematic review generated a concept map for identifying patients requiring highly specialized treatment using non-metric multidimensional scaling and agglomerative hierarchical cluster analysis. The concept map was generated suggested 10 clusters of factors that predicted need for highly specialized interventions. These include severity, onset and treatment course, other psychiatric comorbidities including personality disorders and substance use disorders, somatic comorbidity, unhealthy coping styles, exposure to childhood trauma, social risk factors, and overall psychosocial dysfunction. Such findings highlight the need for comprehensive clinical assessment of patients using both clinical and non-clinical factors (24).

### Treatment and Precision Psychiatry

Despite current knowledge of the heterogeneity of depression, treatments are almost still the same for patients from different clinical profiles. This may explain the finding that roughly 30% of patients with depression recover when using the first prescribed drug (25) and that an estimate of 55% will experience at least one side effect (26). The precision approach may enhance treatment choice as shown in a model involving the specification of a depressive subtype based on symptoms (i.e.: melancholic, psychotic, atypical, and anxious) and making drug choices accordingly. Within each subtype, the model proposes that treatment choice should take into consideration patient-related variables such as personality, personal preferences, comorbidity, and reactions to side-effects. In this regard, treatment is based on a bottom-up approach rather than a generic top-down approach that typically ignores differences in clinical profiles assuming homogeneity (27). Along the same lines, Saltiel and Silvershein (28) called for an adoption of a mechanism-based approach to pharmacotherapy based on the current knowledge of the effects of some drugs on specific clusters of symptoms of depression (anxiety, cognitive impairment, insomnia, hypersomnia, pain, fatigue, psychomotor problems, poor appetite, etc.) that are associated with specific neural circuits. Research also suggests that several factors may explain individual differences in responses to treatment. For example, several findings suggest a link between major depressive disorder and specific microbiome profiles and, similarly, between the use of antidepressants and the gut microbiota being mediated by different forms of antimicrobial activity (29). For example, as one of the most commonly prescribed antidepressants, citalopram is correlated with an increase in the Enterobacteriaceae family (30). Similarly, the use of antidepressants has been correlated with increased bacterial taxa, namely the Helicobacter, Asteroleplasma, Marinilactibacillus genera, Bacillus, and Succinivibrionaceae (31). Additionally, antidepressant pharmacokinetics have proven to differ based on age (32) and sex (33).

The effectiveness of pharmacogenetic-guided treatments in improving clinical outcomes in patients with major depressive disorder and in reducing potential side effects also supports the adoption of a personalized approach. For instance, one randomized controlled trial showed sustained improvement over a 12-week follow-up period and increased side effect tolerability when using pharmacogenetic testing for guiding drug choice (34). In the absence sound empirical evidence supporting the application of such techniques clinically, randomized controlled trials should be encouraged in order to research more genomic information that can foster precision medicine. One study is a prospective randomized controlled trial aiming to assess the effectiveness of pharmacogenetics-guided treatment in cases of depression compared to standard treatments. Using the NeuroIDgenetix^®^ test to identify gene–drug and drug–drug interactions for different medications, results showed significantly higher response and remission rates among patients who received pharmacogenetics-guided treatment (35). Personalized psychiatry has also expanded to include the field of psychotherapy and more research have addressed the application of person-centered therapies in the treatment of depression [see, (36)]. However, more research is warranted in the area of personality psychotherapy, and combined psychotherapy, and pharmacotherapy.

A summary of all studies covered in section Current state of the art in provided in Table 1.

**Table 1:** A summary of studies on genetics, biomarkers, psychosocial markers, and the treatment of major depressive disorder from a personalized perspective.

**Area**	**Authors**	**Design**	**Sample**	**Findings**
Genetics	Bergemann et al. (11)	Integrated knowledge translation	N/A	• Neuropattern can be used as a diagnostic tool based on conceptualized endophenotypes and may have potential in reducing symptomatic severity and improving response to treatment greater reduction in symptomatic severity and faster response to treatment.
	Tansey et al. (12)	Genome-wide complex trait analysis	2,799 patients with MDD treated with antidepressants	• Common genetic variants explained 42% of individual differences in response to antidepressants.
	Bousman and Hopwood (13)	Review	N/A	• Pharmacogenetic tools may prove to be beneficial in improving response to treatment and reducing side effect burden. • There is need for more evidence supporting the clinical use of pharmacogenetics tools. • Pharmacogenetic tools should be utilized only in personal and environmental contexts to demonstrate their clinical utility.
	Hicks et al. (14)	Review	N/A	• Genetic polymorphisms, including CYP2D6 and CYP2C19, can affect response to treatment through their influence on the metabolism of SSRIs.
	Amare et al. (15)	Systematic review of genome wide and candidate gene studies	N/A	• A total of 24 pleiotropic genes hypothesized to be underlie both mood disorders and cardiometabolic diseases risk were identified; *MTHFR, CACNA1D, CACNB2, GNAS, ADRB1, NCAN, REST, FTO, POMC, BDNF, CREB, ITIH4, LEP, GSK3B, SLC18A1, TLR4, PPP1R1B, APOE, CRY2, HTR1A, ADRA2A, TCF7L2, MTNR1B*, and *IGF1*. • Pathways include: corticotrophin-releasing hormone signaling, AMPK signaling, cAMP-mediated or G-protein coupled receptor signaling, axonal guidance signaling, serotonin or dopamine receptors signaling, dopamine-DARPP32 feedback in cAMP signaling, circadian rhythm signaling, and leptin signaling.
	Gandal et al. (16)	Transcriptomic profiling	N/A	• Multiple gene modules could represent cortical dysfunction in different disorders including major depressive disorder. • Major depressive disorder was linked with G-protein coupled receptors, cytokine-cytokine interactions, and hormone activity pathways.
	Feng and Youssef (17)	Review	N/A	• Epigenetics can be used as a clinically valid biomarker predictor of treatment response to ECT.
Biomarkers	Dinan (18)	Review	N/A	• Major depression can be conceptualized as a proinflammatory response marked by increased levels of C-reactive protein and cytokines. • Antidepressants can suppress the inflammatory response. • Electroconvulsive therapy increases levels of proinflammatory cytokines. • A link has been established between depression, inflammation, and cardiovascular risk.
	Pisoni et al. (19)	Cross sectional	36 patients with treatment-resistant depression and 36 healthy controls	• Patients showed lower serum levels of brain-derived neurotrophic factor, vascular endothelial growth factor-C, and higher angiopoietin-1 receptor than healthy controls. • Low VEGF-D levels at admission and low VEGF and VEGFC levels during treatment were linked with non-response to treatment.
Psychosocial markers	Elliott et al. (20)	Review	N/A	• We then consider neuroimaging studies of affective cognition in healthy volunteers, which have led to the development of more sophisticated neural models of these processes.
				• Impairments in affective cognition are significantly linked with mood disorders on both the behavioral and neuroimaginig levels. • Serotonin is hypothesized to be involved in the pathology of depression and to underlie disturbed affective cognition. • Patients with major depressive disorder tend to have significant cognitive biases that contribute to their symptoms in addition to slow responses, poor response to positive stimuli, heightened sensitivity to negative stimuli, and a tendency to make negative interpretations of ambiguous stimuli.
	Godlewska et al. (21)	Clinical trial	35 patients with major depressive disorder besides healthy controls	• Patients with patients showed higher levels of activation to fear facial expressions than healthy controls in the insula and dorsal anterior cingulate. • Responders to treatment had a more significant reduction in neural activity to fearful, rather than happy, facial expressions indifferent brain regions. • Correction of negative bias in patients may underlie response to treatment.
	Shiroma et al. (22)	An 8-week open-label study	27 older subjects with non-psychotic major depressive disorder	• Changes in emotional processing predicted response to treatment.
	Cotter and Barnett (23)	Opinion article	N/A	• Most patients do not reach remission following first antidepressant treatment. • Non-responsiveness to treatment can be linked to poor quality of life, psychology welfare, and a higher financial burden. • A personalized medicine approach may provide solutions to optimize prevention, diagnosis, and bridge the gap between treatment and positive response to treatment. • A focus on cognitive processes with affective components may prove to be of significant clinical utility in mental health treatment. • Cognitive biases are at the core of the pathology of different mood disorders, including major depressive disorder.
	van Krugten et al. (24)	Systematic review and concept mapping	N/A	• A total of 10 major clusters of indicators of need for highly specialized interventions were identified: depression severity, onset and (treatment) course, comorbid personality disorder, comorbid substance use disorder, other psychiatric comorbidity, somatic comorbidity, maladaptive coping, childhood trauma, social factors, and psychosocial dysfunction.
Treatment	Saveanu et al. (25)	Practical trial design	1,008 patients between 18 and 65 years of age	• Following 8 weeks of treatment, 62.2% of patients responded to treatment and 45.4% reached remission on the Hamilton Rating Scale for Depression. • Side effects occurred in 25% or less of the patients with none to minimal levels of intensity and burden.
	Papakostas (26)	Review	N/A	• Side effects related to tolerability may contribute to poor compliance to treatment, increasing risks of relapse. • Clinicians should be familiar with the tolerability profiles of different drugs and should balance the costs and benefits of treatment.
	Bayes and Parker (27)	Narrative review	N/A	• A model based on the depressive subtype or symptom cluster and matching class of antidepressants may help in treatment decisions. • Drug choice within each class can be facilitated based on the patient's personality profile, medical and psychiatric comorbidity and reactions to side effects.
	Saltiel and Silvershein (28)	Review	N/A	• Patients may benefit more from treatments based on deconstructing a diagnosis to symptoms that are targeted specifically to improve functioning and quality of life. • A framework is provided to help clinicians in the provision of personalized pharmacotherapy.
	Walsh et al. (29)	Review	N/A	• The gut microbiome is involved in response to treatment through its effects on drug metabolism, both directly and indirectly. • Drugs also have effects on the function and composition of the gut microbiome with different effects on the host. • A link has been demonstrated between major depressive disorder and specific microbiome profiles and, similarly, between the use of different psychoactive drugs and the gut microbiota.
	Rogers and Aronoff (30)	Cross-sectional	155 adults	• Medications affect the bacterial composition in the gastrointestinal tract depending on the NSAID ingested. • Citalopram is correlated with an increase in the Enterobacteriaceae family.
	Ticinesi et al. (31)	Cross-sectional	76 older inpatients, aged 83 ± 8	• A significant negative correlation has been demonstrated between the number of drugs and number of taxa as measured by the Chao1 Index. • Antidepressants were among the drugs with the highest association with single taxa.
	Boyce et al. (32)	Review	N/A	• Antidepressant pharmacokinetics have shown to differ based on age • Some antidepressants have proven to be linked with significant age-related decline in systemic clearance, including SSRIs, SNRIs, TCAs, and three newer antidepressants.
	Bigos et al. (33)	Review	N/A	• Pharmacokinetics of antidepressants differ by gender which may explain differences in response to antidepressants between sexes.
	Pérez et al. (34)	A 12-week, randomized controlled trial	316 adult patients with major depressive disorder	• Patients in the pharmacogenetics-guided treatment group showed higher response rates to treatment and better tolerability of side effects compared to patients in the control group.
	Bradley et al. (35)	Trial design following aa prospective, randomized	685 patients	• Rates of remission among patients with depression were significantly higher in the pharmacogenetics-guided group than the control group following 12 weeks of treatment.
	Cujipers et al. (36)	Systematic review and meta-analysis of randomized trials	41 studies with 2,741 patients	• Cognitive-behavioral therapy has shown to be more effective in older adults and patients with com orbit addictive disorders than other psychotherapies.

## Challenges in Application

Computer and information technologies have developed over the past years making it easier for research to utilize clinical datasets with high levels of accuracy and relevance, and possibly benefit from artificial intelligence-based modeling systems to predict disease and treatment outcomes. Well-established softwares have been introduced to the precision medicine software market including 2bPrecise, PierianDx, and others. Despite all advances toward adopting a personalized approach in mental health services, and more specifically in the treatment of depressive disorders, several challenges may still limit its clinical utility. One limitation hindering the generalization of such findings to real-life practice is the fact that the majority of clinical trials recruit patients with mild and moderate severities excluding those with more severe symptoms, making them not representative of the actual patient population. Additionally, there is wide disagreement about the validity of categorizing depression into specific subtypes based on symptomatology and the presence of specific endophenotypes (27).

Besides the clinical complexity of depressive disorders, one challenge in precision psychiatry is the technical complexity required to process and analyze multiomics data generated from multidimensional datasets and/or using artificial intelligence modeling. This, despite the availability of health records, may limit the ability to integrate patient information in a clinically relevant manner. Additionally, the variations in the technical means of data collection may be another factor limiting theutility of multiomics data (37). Additionally, the cost-effectiveness of some testing techniques is still not well-established in the first place (38).

Data-driven categorization in clinical practice may help facilitate further screenings and referrals of patients in a cost-effective manner; however, different costs associated with the application of precision psychiatry should also be considered. For example, using new reference paradigms require the appropriate training of healthcare staff in different aspects of precision medicine, including genomics, pharmacogenomics, biomarker in order to allow them to use and interpret generated data. Healthcare and medical research institutions may need to make use of advanced computational systems with additional costs required for the management of data security and privacy, data analysis and interpretation besides the risk of algorithmic failure (37). The costs required for the attainment and analysis of biomarkers and other precision tools may hinder their use in clinical practice, especially in low-income countries (39). This may also mean the presence of health disparities and inequities between patients who may or may not afford precision health services (40).

Various ethical concerns are of utmost importance in relation to precision psychiatry. First of all, protecting the security and privacy of data is challenging and is made more challenging while using electronic health records. Second, the concept of patient stratification, which is at the core of precision medicine, is another substantial concern given the risk of injustice or social discrimination against patients in less privileged groups (i.e., patients with expected poor prognosis) (41), or potentially less profitable patients (i.e., patients of specific socioeconomic backgrounds). Finally, and specific to the field of psychiatry, the use of artificial intelligence in clinical decision making [see (42)] may pose a special concern given the significance of human contact in mental health services.

## Highlight of Future Directions

With more advances in telepsychiatry, research has explored the use of artificial intelligence in moving toward personalized management of mental disorders. For example, technology-based behavioral sensing may prove to be effective in measuring subjective functioning, making inferences about symptoms, and guiding treatment management. For example, self-help tool can be provided through downloadable mobile applications (43). Research does not only support the effectiveness of internet-based interventions in the self-management of depression, but also the symptom-specific effects of such interventions. For example, the well-established Deprexis program that demonstrated effectiveness in terms of symptomatic improvements and low dropout rates has also proven to be specifically effective in alleviating seven specific symptoms of depression in mild and moderate cases (44). Such findings support the need for high specificity in designing automated self-help programs (45).

Functional neuroimaging is another promising tool in guiding treatment decision by providing individual treatment response predictions. Through revealing the pretreatment activation level in some specific neural circuitries, it can be used to predict response to both pharmacological and psychotherapeutic treatments. Patients with higher pretreatment activation level of the ventral and pregenual anterior cingulate cortex (ACC) show better response to antidepressants than to psychotherapy. In light of such findings, functional neuroimaging may ideally guide treatment choice in different subtypes of depression (46). Similarly, a recent systematic review on the use of neuroimagining and behavioral predictors of treatment efficacy identified low baseline responsively in limbic regions of the brain and increased medial and dorsal prefrontal responses to emotional stimuli as the most consistently significant predictors of response to pharmacological treatment for depression (47).

## Author Contributions

RD conceptualized and drafted the work. MS reviewed the work. All authors contributed to the article and approved the submitted version.

## Conflict of Interest

The authors declare that the research was conducted in the absence of any commercial or financial relationships that could be construed as a potential conflict of interest.
